# Age-dependent hormesis-like effects of the synthetic cannabinoid CP55940 in C57BL/6 mice

**DOI:** 10.1038/s41514-020-0045-7

**Published:** 2020-07-06

**Authors:** Erik L. Hodges, Jessica P. Marshall, Nicole M. Ashpole

**Affiliations:** 1 Department of BioMolecular Sciences, Pharmacology Division, University of Mississippi School of Pharmacy, University, Oxford, MS USA; 2 Research Institute of Pharmaceutical Sciences, University of Mississippi School of Pharmacy, University, Oxford, MS USA

**Keywords:** Ageing, Cognitive ageing

## Abstract

Use of cannabis and cannabinoid-containing substances is increasing among geriatric patients, despite relatively sparse preclinical evidence in aged models. To better understand the effects of exogenous cannabinoids on aging male and female rodents, we compared the age- and dose-dependent physiological and behavioral effects of the synthetic cannabinoid CP55940 in young–adult and aged C57BL/6 mice. Locomotion, body temperature, thermal nociception, and fecal output were measured following CP55940 administration. Our findings indicate that CP55940 is more potent and efficacious in older mice, evidenced by exaggerated antinociception and locomotor inhibition when compared to younger adult mice. In addition, we report that low doses of CP55940 paradoxically stimulate locomotion in young–adult (4 m) mice; however, this hormesis-like response is not as evident in aged animals (21–24 m). These bidirectional effects appear to be mediated via the endocannabinoid CB1 and CB2 receptors.

## Introduction

Collectively, the canonical receptors (CB1 and CB2), interacting proteins (cannabinoid receptor interacting protein-1α [CRIP1α]), endogenous ligands (anandamide and 2-arachidonoylglycerol), biosynthetic regulators (N-acyl phosphatidylethanolamine phospholipase D [NAPE-PLD], diacylglycerol lipase-α [DAGLα], and diacylglycerol lipase-β [DAGLβ]), and metabolizing enzymes (fatty acid amide hydrolase [FAAH] and monoacylglycerol lipase [MGL]) of the endocannabinoid system are known to dynamically regulate numerous aspects of mammalian physiology. Though extensive work has been conducted on the behavioral and developmental effects of exogenous cannabinoids early in life, substantially less is known regarding the physiological effects of these substances in advanced age^1,2^. This lack of information, coupled with rapidly shifting trends in cannabis- and cannabinoid-use worldwide puts elderly patients particularly at-risk^3^. Furthermore, emerging evidence suggests that the endocannabinoid system may play a fundamental role in the biological processes of aging. This is exemplified by transgenic mice that are genetically deficient in CB1, which exhibit progeroid cognitive and physiological phenotypes^4^. However, pharmacological manipulation of endocannabinoid signaling does not appear to produce consistent effects across the lifespan. This is evidenced in studies where chronic infusion of the phytocannabinoid Δ-9-tetrahydrocannabinol (THC) was shown to enhance cognition in aged mice at doses which impair younger animals’ performance^5^.

Cannabis and cannabinoid-containing substances have been used therapeutically for millennia, but rigorous evaluation of their medical value is still ongoing^6^. In preclinical rodent models, the effects of CB1-acting compounds are traditionally measured using the “Tetrad Assay”—a battery of tests that measure locomotion (open field), body temperature (rectal probe), catalepsy (ring-immobility), and nociception (hot plate or tail withdrawal)^7,8^. The synthetic cannabinoid CP55940 is a well-documented, high-affinity CB1/CB2 mixed agonist, which elicits the hallmark behaviors tested in these assays. Studies have demonstrated that the behavioral effects of CP55940 are similar to those produced by high doses of the phytocannabinoid THC—hypolocomotion, hypothermia, and antinociception—though CP55940 is substantially more potent and efficacious than THC in these assays^9^.

While there is consensus regarding the depressant, aversive, and antinociceptive effects of CB1 agonists at high doses, several studies report contradictory stimulation of locomotion, body temperature, and cognitive behaviors at low or ultralow doses^1,10–12^. One explanation for these biphasic dose responses is the biological adaptive response known as *hormesis*^12,13^. As defined by Calabrese and Baldwin, *hormesis* refers to the manner in which biological systems adapt to slight environmental perturbations and subsequently alter their performance^14^. In the pharmacological context, hormesis may manifest as a nonlinear or bidirectional dose-response, in which low amounts of a substance produce the opposite effects of higher doses. Though the extent of low-dose stimulation in these systems is reported to be modest (30% of vehicle-treated response), the therapeutic application of pharmacological hormesis in a manner similar to preconditioning might enhance the resilience of aging systems^13^.

Hormesis, preconditioning, and bidirectional dose responses are widely evident in numerous biological systems, though systematic evaluation of these modest low-dose effects is difficult and therefore often overlooked. Nevertheless, these findings are critical, as hormesis-like effects may rectify conflicting results observed between treatment groups. Regarding exogenous cannabinoids, several lines of evidence demonstrate that treatment with low doses of these substances benefits cognition in aged animals; however, administration of these same doses to younger animals results in cognitive impairments^12,15,16^. Hormesis-like responses to cannabinoids have been reported by several studies using young animals, though it remains to be seen whether or not this hormetic responses is consistent throughout the lifespan^1^. To test whether the bidirectional, hormesis-like response to cannabinoids is age-dependent, we experimentally assessed the dose-dependent physiological and behavioral effects of CP55940 at multiple doses in young–adult (4 m) and aged (21–24 m), male and female mice.

## Results

### Acclimation to vehicle injection and baseline

In order to adequately capture potential biphasic behavioral and physiological responses elicited by the synthetic CB1/CB2 agonist CP55940, we administered this compound via intraperitoneal (IP) injection at the following weight-adjusted doses: 0.001, 0.01, 0.1, 0.4, and 0.8 mg/kg. Prior to treatment with CP55940, animals underwent a 6-day acclimation period consisting of 3 days of handling and temperature measurement, followed by 3 additional days of temperature recordings before and after vehicle injection (1:1:18, EtOH:Kolliphor:Saline) (Supplementary Fig. 1). During the acclimation period, baseline temperature was measured immediately before and 60 min after injection using both a rectal probe and a non-contact infrared (IR) thermometer. During acclimation recordings, both young–adult (4 m) and old (21–24 m) mice showed a slight but consistent average reduction in rectal and IR temperatures 60 min after injection of the vehicle solution (Supplementary Fig. 2). In addition, old mice weighed more while having lower baseline rectal and skin/pelage temperatures than young–adult mice (Supplementary Fig. 2).

### Behavioral and physiological responses to CP55940

**Fig. 1:**
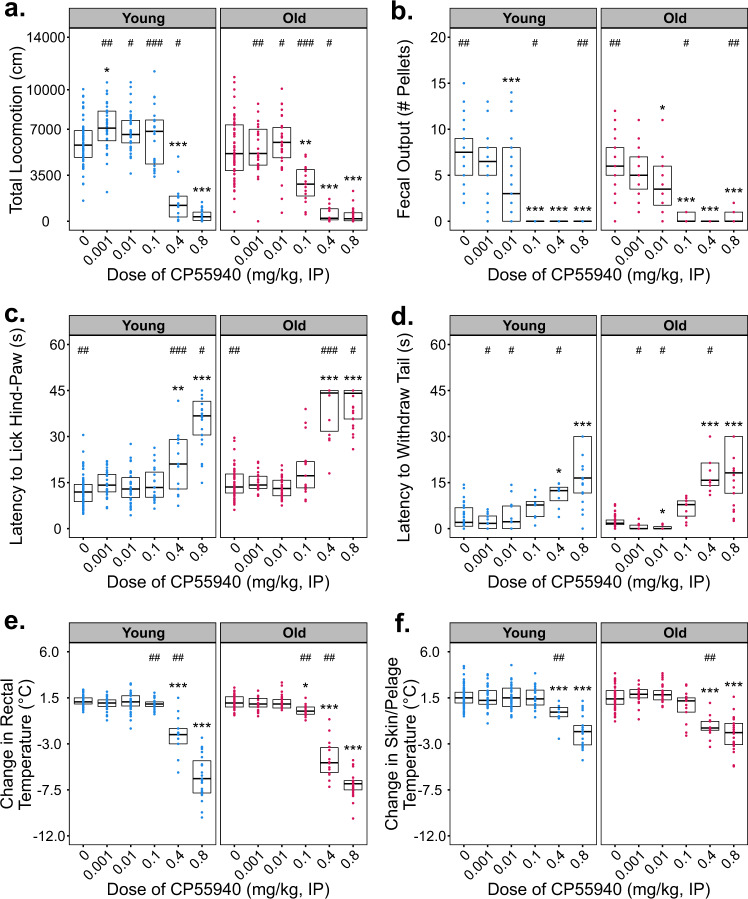
Effects of CP55940 on locomotion, thermoregulation, and nociception in Young–adult (4 m) and old (22 m) C57BL/6 mice. CP55940 was administered via intraperitoneal injection at doses ranging from 0.001 to 0.8 mg/kg to young and old male and female mice. Individual data points for each testing group are overlaid on Tukey-style boxplots. The lower and upper edges of each box represent the first and third quartiles, respectively. The middle horizontal line in each box depicts the median of that dose/age group. Sample sizes for every treatment group and figure panel are listed in Supplementary Fig. 5. Statistical comparisons of age, dose, and age:dose interactions were made using the nonparametric Scheirer–Ray–Hare test. Significant omnibus results were followed by planned post hoc contrasts of each dose vs. vehicle-treated control groups using Dunn’s method. The false discovery rate was corrected for using the Benjamini, Hochberg, and Yekutieli method. Within an age group, significant differences (*P* < 0.05) between a given dose and the vehicle response (0 dose) are indicated using asterisks (*). Differences between age groups at a given dose are indicated by pound signs (#). **a**, **b** Total locomotion and fecal output during open-field testing, 30–60 min after injection of CP55940 or vehicle. **c** Latency to exhibit nociceptive behavior when place on a 52 °C hot plate. **d** Latency to withdraw the tail from 46 °C water. **e** Change in rectal temperature 60 m after injection of drug. **f** Change in infrared recording of skin/pelage temperature 60 m after injection of drug.

On the day of behavioral testing, male and female mice were injected with varying doses of CP55940 or vehicle then assessed for locomotor, thermoregulatory, or nociceptive responses. No sex-specific differences were observed throughout the study, thus the data from both males and females were combined. Activity measurements recorded in the open field 30–60 min after injection indicate that treatment with CP55940 displays a significant age:dose interaction effect on total locomotion (locomotion~age × dose, H(5351) = 11.451, *P* = 0.043). Interestingly, young–adult mice treated with the lowest dose of CP55940 tested (0.001 mg/kg) exhibit significantly increased locomotion compared with age-matched vehicle-treated control animals (*P* < 0.05, Fig. 1a). However, old animals administered this same dose did not significantly differ from age-matched vehicle-treated controls. As expected, higher doses of CP55940 significantly reduced locomotion in both young–adult and aged animals, though this suppression was evident in aged animals at a dose of 0.1 mg/kg, which did not inhibit locomotion in young–adult animals (Fig. 1a). In both age groups, doses of CP55940 ≥ 0.4 mg/kg induced severe catalepsy (Fig. 1a). Though no effect of age was detected on fecal output during the open-field task (feces~age, H(1297) = 1.57, *P* = 0.210), young vehicle-treated animals produced significantly more fecal pellets during the open-field task than old control animals (*P* = 0.004). In addition, at doses of CP55940 ≥ 0.01 mg/kg fecal output was significantly reduced in both age groups (Fig. 1b).

Immediately following the 60-min temperature recordings, nociception was quantified by placing each mouse onto a 52 °C heated plate, and recording the latency to lick its hind-paw or jump. Significant simple main effects of dose (latency~dose, H(5353) = 149.41, *P* < 0.001) and age (latency~age, H(1353) = 9.21, *P* = 0.002) were detected in this assay, though no interaction between these factors was observed (Fig. 1c). Among vehicle-treated animals, young mice were significantly quicker to respond than older mice (*P* = 0.005). The antinociceptive effects of CP55940 were highly significant in both age groups at doses of 0.4 and 0.8 mg/kg, though aged animals were significantly slower to respond than young animals at both of these doses. In later cohorts of animals, nociception was also assessed via tail-immersion in a hot-water bath (46 °C). The results from these tests were similar to the hot plate, although no main effect of age was detected (Fig. 1d). Intriguingly, a significant reduction in latency to withdraw tail was observed in old animals treated with the intermediate dose of 0.01 mg/kg (*P* = 0.049). In agreement with the hot plate recordings, age groups showed highly significant antinociception at doses of CP55940 ≥ 0.4 mg/kg.

Rectal and skin/pelage temperatures were measured 60 min post injection via rectal probe and infrared thermography. Both of these measures displayed a significant main effect of dose during behavioral testing (Δrectal~dose H(5349) = 182.01, *P* < 0.001 and Δskin~dose, H(5340) = 146.06, *P* < 0.001). In vehicle-treated animals, rectal and skin/pelage temperatures were significantly elevated from baseline in young–adult and old mice (Fig. 1e, f). When compared with vehicle-injected animals, high doses of CP55940 (0.4 and 0.8 mg/kg, IP) significantly reduced rectal temperature in both age groups. Importantly, this decline in rectal temperature was more pronounced in aged mice, which exhibited reductions in rectal temperature at the dose of 0.1 mg/kg which had no effect in younger animals.

### Responses to CP55940 and CB1 or CB2 antagonism

**Fig. 2:**
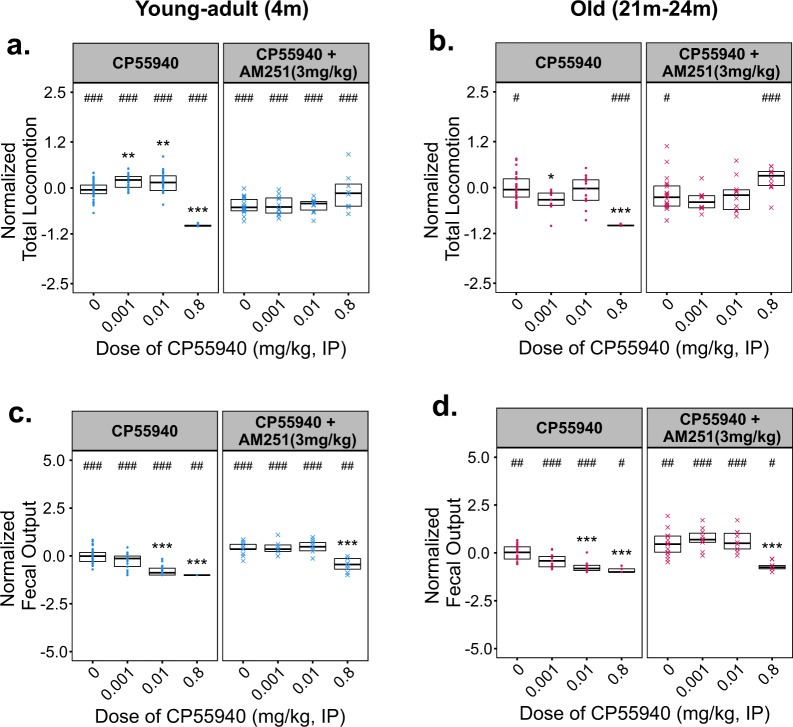
Effects of CP55940 Co-administered with the CB1 inverse agonist AM251 on locomotion and fecal output in young–adult (4 m) and old (22 m) male C57BL/6 mice. Varying doses of CP55940 were administered alone or in a pre-mixed solution with 3 mg/kg of AM251 to young–adult and old male mice. **a**, **c** Total distance moved (**a**) and total fecal output (**c**) by young–adult (4 m) mice during 30 m of open-field testing. **b**, **d** Total distance moved by old (22 m) mice during 30 m of open-field testing. All data have been normalized to the responses of vehicle-treated age-matched control animals which were tested at the same time. Individual data points for each testing group are overlaid on Tukey-style boxplots. The lower and upper edges of each box represent the first and third quartiles, respectively. The middle horizontal line in each box depicts the median of that dose/age group. Sample sizes for every treatment group and figure panel are listed in Supplementary Fig. 5. Significant omnibus results were followed by planned post hoc contrasts of each dose vs. vehicle-treated control groups using Dunn’s method. The false discovery rate was corrected for using the Benjamini, Hochberg, and Yekutieli method. Within an age group, significant differences (*P* < 0.05) between a given dose and the vehicle-treated response (0 dose) are indicated using asterisks (*). Differences between CP55940 alone and CP55940 + AM251 at a given dose are indicated by pound signs (#).

**Fig. 3:**
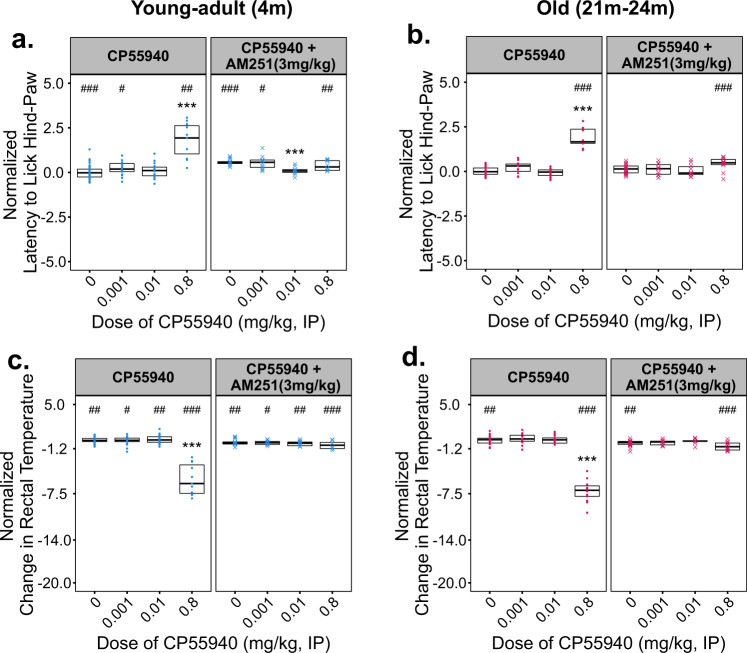
Effects of CP55940 co-administered with the CB1 inverse agonist AM251 on nociception and thermoregulation in young–adult (4 m) and old (22 m) male C57BL/6 mice. Varying doses of CP55940 were administered alone or in a pre-mixed solution with 3 mg/kg of AM251 to young–adult and old male mice. **a**, **b** Latency to lick the hind-paw of young–adult (**a**) and old (**b**) male mice when tested on the hot plate 60 m after drug injection. **c**, **d** Change in rectal temperature of young–adult (**c**) and old (**d**) male mice 60 m after drug injection. All data have been normalized to the responses of vehicle-treated age-matched control animals which were tested at the same time. Individual data points for each testing group are overlaid on Tukey-style boxplots. The lower and upper edges of each box represent the first and third quartiles, respectively. The middle horizontal line in each box depicts the median of that dose/age group. Sample sizes for every treatment group and figure panel are listed in Supplemental Fig. 5. Significant omnibus results were followed by planned post hoc contrasts of each dose vs. vehicle-treated control groups using Dunn’s method. The false discovery rate was corrected for using the Benjamini, Hochberg, and Yekutieli method. Within an age group, significant differences (*P* < 0.05) between a given dose and the vehicle-treated response (0 dose) are indicated using asterisks (*). Differences between CP55940 alone and CP55940 + AM251 at a given dose are indicated by pound signs (#).

To determine whether the biphasic effects of CP55940 could be accounted for by activity at canonical cannabinoid receptors, we co-administered CP55940 with the CB1 or CB2 inverse agonists—AM251 or AM630, respectively. Based on previous literature, doses of 3 mg/kg AM251 or AM630 were administered to modulate CB1- and CB2-mediated signaling and behavior^17,18^. Two doses of CP55940 shown to stimulate locomotion in young males (0.001 and 0.01 mg/kg, IP) were administered alone or in the presence of AM251 or AM630; in addition, a high dose (0.8 mg/kg) that significantly reduces locomotion in both young and old animals was co-administered with AM251 (3 mg/kg). AM251 alone significantly reduced locomotion and attenuated both the stimulatory and inhibitory effects of CP55940 on locomotion in young–adult males (Fig. 2a). In contrast, AM630 coadministration in young males significantly attenuated the psychomotor stimulation elicited by CP55940, but did not block the reduction in fecal output produced by CP55940 at a dose of 0.01 mg/kg (Supplementary Fig. 3). The CB1 inverse agonist AM251 also blocked the inhibitory effects of 0.01 mg/kg CP55940 on fecal output in young males (Fig. 2c). Fecal output was significantly increased following coadministration of AM251 with 0.8 mg/kg CP55940 compared with 0.8 mg/kg CP55940 alone; although this was a partial rescue by AM251, as a significant decrease from vehicle control was still observed during coadministration of the drugs. Similar to the rescue of locomotor inhibition, the antinociceptive and hypothermic effects of 0.8 mg/kg CP55940 were abrogated by 3 mg/kg AM251 in young and old mice (Fig. 3a–d), suggesting CB1 mediates these responses to high-dose synthetic cannabinoid administration.

While aged animals did not show significant stimulatory locomotor effects with low doses of CP55940, we verified that the mechanism responsible for the changes observed with high doses of CP55940 was unchanged in advanced age. Coadministration of CP55940 with AM251 significantly attenuated the hypolocomotor (Fig. 2b), antinociceptive (Fig. 3b), and hypothermic effects (Fig. 3d) of CP55940 in aged males. Similar to the effects in young–adult males, changes in fecal output were partially rescued by coadministration of AM251 with CP55940 in aged males (Fig. 2d), although it is important to note that 3 mg/kg of AM251 alone significantly increased fecal output (Fig. 2d). AM251 also reduced locomotor activity and significantly reduced body temperature in the aged mice (Figs. 2b and 3d), similar to the changes seen with AM251 alone in the young males (Figs. 2a and 3c).

### Pharmacokinetics of CP55940 and endocannabinoid gene expression in the brain

To determine the mechanism for the enhanced potency of CP55940 seen in aged animals, pharmacokinetic distribution of CP55940 in the blood and brain was quantified following either 0.1 or 0.8 mg/kg injections. Unfortunately, both of these efficacious doses fell below the LC-MS/MS detection limits in these samples (data not shown). To determine whether age-related changes in endocannabinoid gene expression occur within the mouse cortex and hypothalamus, we performed qPCR on the following endocannabinoid targets: CB1 (*Cnr1*), CB2 (*Cnr2*), CRIP1α (*Cnrip1α*), and FAAH1 (*Faah1*). Compared with young animals, significant reductions in CB1, CRIP1α, and FAAH1 gene expression were observed in aged cortical (*P* = 0.034, 0.047, *P* = 0.002, respectively), but not aged hypothalamic tissue samples (Table 1). No significant differences in CB2 (*Cnr2*) gene expression were detected in either cortical or hypothalamic tissues.

**Table 1:** Quantitative PCR analysis of gene expression in regions of brain tissue from young–adult (4 m) and old (22 m) male mice.

Cortex	*n*	CB1 ± SE	CB2 ± SE	CRIP1 ± SE	FAAH1 ± SE
Young	6	1.00 ± 0.09	1.00 ± 0.04	1.00 ± 0.22	1.00 ± 0.09
Aged	5	0.81 ± 0.13	0.78 ± 0.20	0.63 ± 0.15	0.59 ± 0.09
		*P = 0.034	*P* = 0.239	*P = 0.047	*P = 0.002

Hypothalamus	*n*	CB1 ± SE	CB2 ± SE	CRIP1 ± SE	FAAH1 ± SE
Young	6	1.00 ± 0.24	1.00 ± 0.46	1.00 ± 0.20	1.00 ± 0.17
Aged	5	0.49 ± 1.30	2.55 ± 0.47	1.09 ± 0.71	0.75 ± 1.03
		*P* = 0.12	*P* = 0.06	*P* = 0.52	*P* = 0.15

Delta–delta CT was calculated using HPRT as the housekeeping control. Gene expression values for old mice have been normalized to the young group.

*P ≤ 0.05 *t* test, young vs aged.

## Discussion

Taken together, these findings reveal that the effects of CP55940 on thermoregulation and behavior are highly age- and dose-dependent. Importantly, CP55940 given to aged mice is significantly more efficacious than equivalent doses administered to young mice, and the dose of 0.1 mg/kg appears to be a key inflection point in this differential response. In young mice, our data indicate that low (<0.01 mg/kg, IP) and high (>0.1 mg/kg, IP) doses of CP55940 bidirectionally regulate measures of locomotion, and these increases in total locomotion are not associated with thigmotactic aversive behavior (Supplementary Fig. 6)^19^. However, under the present conditions we did not detect significant CP55940-induced stimulation of locomotor activity in aged mice at any dose of CP55940 tested. The distinct locomotor responses observed in both age groups at doses of 0.1 mg/kg CP55940 indicate that continued characterization of this specific dosing window may help to clarify these age-dependent effects^12,15^.

In contrast to the antinociceptive effects of CP55940 at doses ≥0.4 mg/kg, aged mice demonstrated significantly enhanced nociception at a single intermediate dose (0.01 mg/kg) in the hot-water tail-withdrawal task (Fig. 1d). Although the hot plate and tail-withdrawal tasks both measure nociception, the tail-withdrawal reflex is primarily generated within the spinal cord^20^, while the behaviors elicited by the hot plate are generated supraspinally^21^. In addition, the hind-paw licking measured in the hot plate task requires substantially greater motor coordination to evoke. Therefore, the observation that old mice exhibit enhanced nociception at the single dose of 0.01 mg/kg can be explained by differences in spinally mediated cannabinoid reception or the enhanced sensitivity of the tail-withdrawal task. The ability to detect this effect highlights the importance of selecting tasks with wide detection ranges and sensitivity when studying hormesis. Moreover, the age-dependent responses seen in these assays of nociception further demonstrate the importance of using multiple endpoints when studying aged animals. Given the high prevalence of elderly patients seeking cannabinoid-based therapeutics for chronic pain, additional studies in these models are greatly needed.

During extensive vehicle acclimation recordings, both young–adult (4 m) and old (21–24 m) mice exhibited slight reductions in core-body and skin/pelage temperatures 60 min post injection (Supplementary Fig. 2). However, on the day of behavioral testing, vehicle-treated animals consistently showed increases in rectal and IR temperatures (Fig. 1d, e). One explanation for this differential response is that during behavioral testing, postinjection temperatures were taken immediately after animals completed open-field locomotor assessment. Therefore, it is possible that the hyperthermia observed following vehicle-treatment in this context is confounded by either locomotion or stress-induced hyperthermia from open-field testing. Furthermore, the high rectal temperatures recorded in young vehicle-treated mice following open-field testing may represent a ceiling-effect, precluding the replication of previously observed hyperthermia induced by low doses of cannabinoids^11^. As previously reported, the effects of cannabinoids on thermoregulation may not be limited to brain-stem-mediated mechanisms and may also be influenced by peripheral vasodilation^22^. Thus, we chose to measure skin temperature in addition to core-body temperature. A reduction in temperature of both measures was noted with high doses of CP55940 in all groups tested.

Consistent with previous studies, no significant differences in locomotion were observed with AM630 alone in this study (Supplementary Fig. 3). However, we were surprised that the psychomotor stimulation with low doses of CP55940 was blocked by either AM251 or AM630. This suggests that both CB1 and CB2 mediate, at least in part, the hormetic-like response. Future studies comparing the effects of CB1 and CB2 antagonists using cognitive assays where ultralow doses of cannabinoids have demonstrated protective effects might further delineate whether the observed blockade of hormesis is unique to locomotion or extends to other behaviors in rodents.

It is not currently clear what the mechanism is which increases responsiveness to this cannabinoid in the aged mice. In this study, we observed significantly decreased CB1 (*Cnr1*), CRIP1α (*Cnrip1α*), and FAAH1 (*Faah1*) gene expression in cortical tissue samples from aged mice. This is similar to previous research which demonstrated CB1 levels vary throughout the lifespan, although these age-related changes appear to be region-specific as we did not detect significant gene expression differences in the hypothalamus^23,24^. A reduction in receptor expression by aged animals that show enhanced CB1-dependent physiological behaviors is perplexing, though there is well-established precedence in other neurotransmitter systems that signaling can be enhanced in a compensative manner following chronic receptor blockade^25^. Based on the data we collected, reductions in the expression of FAAH1 (*Faah1*) in the aged cortex suggest that endocannabinoid agonist levels are elevated in our aged mice. Coupled with the observed age-related reductions in CRIP1α (a repressor of constitutive CB1 activation^26^), these two expression-level changes may compensatively elevate cortical CB1 signaling in the aged mouse brain (Table 1). Future studies of cannabinoid receptor heterodimerization and biased agonism, which have yet to be assessed in aged mammals, may also yield beneficial insight on this matter. In addition, cannabinoids may have increased access to the central nervous system due to declining blood brain barrier function in aged animals. During the course of this study, we attempted to assess this hypothesis by measuring the pharmacokinetic distribution of CP55940 in two independent cohorts of young and aged mice following injections of either 0.1 mg/kg or 0.8 mg/kg. Despite the fact that these doses consistently elicited behavioral effects that significantly differed from vehicle-treated mice, this amount of CP55940 ultimately proved to be below our detection limits in serum, liver, and whole-brain tissue samples.

Mechanistically, our studies with the CB1 and CB2 inverse agonists suggest that the psychomotor, thermoregulatory, and antinociceptive effects of CP55940 at doses of 0.001 and 0.01 mg/kg, IP are independently mediated by both receptors. Treatment with AM251 alone produced simultaneous reductions in locomotion and rectal temperature, while greatly increasing fecal output, suggesting that AM251 at a dose of 3 mg/kg,IP is aversive in young male mice. The notable observation that CP55940 at 0.01 mg/kg abrogates the antinociceptive effects of AM251 in young males (Fig. 3a) suggests that this dose of CP55940 in combination with AM251 may restore tonic endocannabinoid activity to baseline levels. The importance of maintaining tonic endocannabinoid signaling has been discussed in detail previously, and the limited effect of AM251 administration, by itself, suggests that the age-dependent responses we observed may be due to elevated endocannabinoid tone^27^. As discussed, our aged animals exhibited significant decreases in FAAH1 and CRIP1α (Table 1). These results suggest that both increased anandamide levels and CB1 may signaling occur in the aged cortex. Taken together, these findings may partially explain the enhanced responses and baseline differences observed between age groups (Fig. 1c). The fact that CB1 antagonism rescues many of the behavioral changes observed with high doses of CP55940 in both the young and old mice supports a substantial role for this signaling pathway throughout the lifespan. However, the failure of AM251 coadministration to rescue the reduction in fecal output seen at the highest dose (0.8 mg/kg, IP) of CP55940 tested (Fig.2c, d) suggests a CB1-independent mechanism, and may be due to interactions with the TRPV1 receptor as recently reported^28^.

We were surprised that the effects of CP55940 were not sex-specific, as studies of either aging or cannabinoids often report pronounced sex-dependent behavioral profiles^29–32^. Moreover, studies of nociception and pain responses also commonly show sex-dependent effects^33,34^, as well as known sex-specific differences in cannabis abuse and physiological responses following cannabis consumption in humans^35,36^. These changes are hypothesized to be driven, in part, by differences in muscle mass and fat tissue distribution. In this study, we did not control for changes in body composition, estrous state, or the stage of reproductive senescence within our young or aged cohorts, but weight was consistently recorded and all drugs were administered at weight-adjusted doses (Supplementary Fig. 2). Our data suggest that the psychostimulatory effects at low doses and robust inhibitory effects at high doses of this synthetic cannabinoid are more strongly influenced by age than sex in mice.

This study offers substantial evidence that the behavioral and physiological effects of CP55940 are highly age-dependent, though several additional considerations remain to be addressed. Specifically, the current experiments were restricted a highly potent synthetic cannabinoid known to agonize both CB1 and CB2. Despite its high affinity for these receptors, high doses of CP55940 have been shown to modulate other receptors and effect physiological functions in cells that lack CB1 or CB2^37^. The expansive endocannabinoidome presents numerous additional targets, such as the previously mentioned TRPs or biosynthetic/metabolizing enzymes, which are also likely involved in mediating the diverse effects of CP55940^38^. In addition, though the consumption of synthetic cannabinoids is increasing^39^, the use of cannabis and isolated phytocannabinoids is far more common. Many phytocannabinoids exhibit unique pharmacological profiles that target both canonical CB1 and CB2 endocannabinoid receptors as well as other nonclassical endocannabinoid targets^38^. Accordingly, expanded studies on the age-dependent effects of cannabis-derived compounds such as THC and non-psychoactive constituents like CBD and terpenes are now greatly needed.

Rising healthcare costs coupled with the increased availability of cannabinoid-based substances marketed as therapeutics have put elderly patients in a particularly vulnerable position^3^. Although the aging process in humans and rodents is multifaceted and exceedingly diverse, reduced physical activity, sleep disturbance, and chronic pain are some of the most commonly reported conditions in advanced age. This study indicates that in mice, the synthetic cannabinoid CP55940 exhibits bidirectional and behavior-specific effects that are greatly influenced by the age of the subject. Therefore, additional studies are required to discern the mechanistic basis for this increased responsiveness in aged rodents and to determine whether these age-dependent effects are species and/or compound-specific. In the wake of rapidly shifting policies affecting access to cannabinoids worldwide, additional preclinical evidence in aged models will be vital to anticipate potential effects these substances may have on aging populations.

## Methods

### Animals

All procedures were approved by the University of Mississippi Institutional Animal Care and Use Committee (IACUC). Experimental cohorts consisted of two age groups: 2–4 months (young–adult) and 21–24 months (old) of age. Experiments were performed on male and female C57BL/6NHsd mice acquired from Envigo (Cat. #: 044) or C57BL/6 mice from the National Institutes of Aging Aged Rodent Colony. A priori power analysis indicated that statistically significant differences based on a medium effect (Cohen’s f > 0.25) could be detected with group sizes *n* > =10. The total number of animals used for this study is listed in Supplementary Fig. 4. All animals were housed in the AALAC-accredited University of Mississippi Animal Facility and given access to food (Cat. #: 7001, Envigo Teklad 4% Fat Rodent Diet) and tap-water ad libitum for the duration of experiments. Mice were group-housed (four mice/cage) in climate-controlled rooms (30–40% relative humidity, 21–23 °C) under a 12:12 “reverse” light cycle (lights OFF at 8:00am and ON at 8 pm). Upon arrival into the facility, animals were immediately group-housed in 75 in^2^ clear polycarbonate cages (Cat. #: AN76, Ancare) with 1/8” corn-cob bedding and environmental enrichment (Cotton Nestlets, Ancare, and Crink’l Nest, The Andersons Lab Bedding) then allowed to acclimate to the facility for 7 days before experimentation. Following this week of acclimation, animals were weighed, fitted with a metal ear tag (Cat. #: INS1005-1LSZ, Kent Scientific) and randomized into balanced experimental treatment groups based on body weight. Mice were checked daily, and clean cages were replaced every week. All experiments and procedures were performed under dim red light (<1 lux) during the animals’ active period.

### Blinding

The experimenter was blinded to all treatment groups during drug administration and experimentation through the use of arbitrarily coded drug containers.

### Drugs, dosing, and administration

The synthetic cannabinoid CP55940 (Cat. #: 0949, Tocris) was initially diluted in 100% ethanol (Cat. #: 2701, Decon Labs) to a stock concentration of 10 mg/mL and kept at −20 °C. The CB1 antagonist, AM251 (Cat. #: 1117, Tocris) was initially diluted in 100% ethanol (Cat. #: 2701, Decon Labs) to a stock concentration of 10 mg/mL and kept at −20 °C. The CB2 antagonist, AM630 (Cat. #: 1120, Tocris) was initially diluted in 66.6% ethanol (Cat. #: 2701, Decon Labs) and 33.3% dimethyl sulfoxide (DMSO, Cat. #: 276855, Sigma-Aldrich) to a stock concentration of 6.66 mg/mL and kept at −20 °C. On the day of each experiment, fresh vehicle solution was prepared by first dissolving Kolliphor EL (formerly known as Cremophor EL, Cat. #: C5135-500G, Sigma Life Science) in 100% ethanol, then adding sterile 0.9% HSP pH 7.4 saline (Cat. #: 00409488810, Hospira) in a ratio of (1:1:18). Experiments using AM251 or AM630 were performed by pre-mixing either AM251 or AM630 with respective doses of CP55940 prior to injection. All drug mixtures were administered at a volume of 1 mL/100 g body weight.

During testing, the animal’s temperature and weight were recorded then a body weight-adjusted dose of vehicle or CP55940 was administered via intraperitoneal (IP) injection using a 1 mL syringe (Cat. # 309602, Becton, Dickinson and Company [BD]) and a 25 gauge needle (0.5 × 16 mm, Cat. # 305122, BD).

### Acclimation

On the day of behavioral testing, all animal cages were placed onto a plastic or metal cart and relocated to the testing room where they were allowed to acclimate for 3 h prior to testing. Throughout the acclimation and testing periods, the room remained dark and white noise was continuously played at a volume of ~60 db.

### Body weight

Body weight was measured at the start of every experimental procedure to ensure accurate drug administration and as an endpoint for exclusion in the event that an animal’s mass decreased >10% in a week. Animals were gently removed from their cages by lightly gripping the base of their tail and placed into a lidless pipette-tip box on top of the balance. Once the animal ceased, moving the mass was recorded. In between recordings, the pipette-tip box was wiped clean with ethanol, dried with a paper towel, and the balance was tared.

### Rectal and pelage/surface temperature

Rectal temperature was measured using a Digi-Sense Advanced Precalibrated Thermocouple Thermometer (Cat. #: EW-20250-91, Kent Scientific) which was connected to a 1.9 cm × 0.165 cm rectal probe (Cat. #: RET-3, Kent Scientific). Before measurement, the rectal probe was cleaned using 70% ethanol, dried, and lightly lubricated with petroleum jelly. Animals were briefly immobilized on the top of a metal cage lid, and then securely restrained by gripping the scruff (nape of the neck). After confirming the animal’s identification/ear tag, the rectal probe was gently inserted 1.9 cm into the animal’s rectum. Once a stable measurement was achieved (~3–5 s), the temperature was recorded.

Skin/pelage temperature was measured via infrared thermography at the dorsal pelvis using a FLIR Non-Contact Infrared Spot Thermal Camera (Cat. #: TG165, FLIR). Emissivity was set to 0.85, as suggested previous research^40^. Immediately after weighing, while the animal was standing still and upright in an empty pipette-tip box, the thermal camera was held directly above the animal exactly 10 cm away from the animal’s skin. Once a stable measurement was achieved (~3–5 s), the temperature was recorded.

### Locomotion—open field

Locomotor activity was measured by placing a single mouse into a lidless clear-plastic open-field arena (41 × 41 × 38 cm), which was surrounded by a 16 × 16 photobeam array (Photobeam Activity System, San Diego Instruments) or recorded using a ceiling-mounted camera. Locomotion in the *X* and *Y* directions were quantified using beam breaks or software-based tracking (Ethovision XT-13, Noldus). During recording, four open-field arenas were used simultaneously, and each arena was shielded on three sides by 60-cm black plastic dividers so that animals could not see each other. When administered at high doses, the psychoactive effects of CP55940 are pronounced from 10 to 120 min. Thus, locomotor activity was measured continuously for 30 min, beginning 30 min after drug or vehicle injection. Beam breaks in the *X* and *Y* directions were sampled every 5 s for 30 min and then exported to spreadsheets at the conclusion of testing. Peripheral locomotion was defined as any locomotion which occurred in the outermost 50% of the open-field arena. After removal from the open field, animals were briefly (1–5 min) placed into temporary cages before testing on the hot plate.

### Nociception—hot plate

Nociception was measured by placing a single mouse on top of a preheated 52 °C thermal block (Cat. #: PE34, IITC Life Sciences), and recording the latency to withdraw and/or lick their hind paws. During testing, a hollow clear-plastic cylinder 25 cm in diameter by 35 cm high was placed on top of the heated thermal block to prevent animals from leaving the hot plate. Animals were placed in the cavity of the plastic cylinder directly in contact with the hot plate and closely monitored for nociceptive responses. Based on preliminary experiments, we concluded that the latency to lick the hind paws was the most reliable indicator of nociception. Immediately after licking their hind paws, the timer was stopped and the animal was removed from the hot plate and placed back into a temporary cage. If an animal failed to respond to the thermal stimulus in 45 s, the timer was stopped and the animal was rapidly removed from the hot plate to prevent tissue damage.

### Nociception—hot-water tail withdrawal

Several cohorts of male and female mice were also tested using a hot-water bath preheated to 46 °C. After testing on the hot plate, animals were allowed a 90-s recovery period, then gently scruffed by the nape of the neck and their tails were dipped 1/3 from the tip into heated water. The latency to withdraw the tail was manually recorded using a stopwatch.

### Gene expression—qPCR

Cortical and hypothalamic RNA was isolated using the RNA-easy mini kit (Qiagen) and converted to cDNA using High-Capacity RNA-to-cDNA kit (Applied Biosystems). RT-PCR was performed using TaqMan Universal PCR Master mix (Life Technologies) and TaqMan validated primers (Mm01212171_s1 (*Cnr1*), Mm02620087_s1 (*Cnr2*), Mm01187898_m1 (*Cnrip1α*), Mm00515684_m1 (*Faah1*), Mm03024075_m1 (*Hprt*)) on the CFX connect real-time PCR detection system (Bio-Rad). Delta–delta CT was then calculated using HPRT as the housekeeping gene. The experimenter was unaware of age group during testing, and was unblinded during delta–delta CT analysis.

### Experimental timeline

After ear-tagging and enrollment into treatment groups, a 6-day acclimation period was begun. Initially, animals were acclimated for 3 days to handling, weighing, and temperature measurement with the rectal probe and IR camera. Next, the animals were acclimated for 3 days to all of the previous procedures, as well as IP injection of vehicle (1:1:18, Ethanol:Kolliphor EL:Saline) and a follow-up 60 m temperature recording after the initial injection. Drug administration and behavioral experiments were performed on the 7th day. In an effort to reduce animal number, mice were given a wash-out period of 7 days and reenrolled into the study design using a Latin square approach. (Supplementary Fig. 2). Subsequent rounds of testing used only a 3-day acclimation period, wherein the animals receive vehicle injections and 60 m follow-up temperature recordings. On the day of re-testing, animals were randomly assigned to doses which they had not previously received and the experimenter was again blinded to the treatment groups.

### Data analysis, statistics, and exclusions

Data analysis was performed, collected, and analyzed using a combination of Microsoft Excel (v2016, Microsoft), RStudio (v.1.1.456, RStudio Team), MATLAB (r2019a, MathWorks), PAS (v2.0.3, San Diego Instruments), PAS Data Reporter (v1.0.1.0, San Diego Instruments), Ethovision XT (v13, Noldus), and G*Power (v3.1^41^).

Prior to omnibus testing, all data for a given measurement were tested for outliers, which was defined as those points that were greater or less than three interquartile ranges above or below the third and first interquartile ranges within a single-treatment group. After exclusion of these points, which are listed in Supplementary File 1, data from each measurement were assessed for a normal distribution (Shapiro–Wilk test) and equal variance (Levene test). Since all measures observed failed these tests, simple main effects and interactions were assessed using the nonparametric Scheirer–Ray–Hare (S–R–H) test. A priori significance was set at α = 0.05. Significant main effects detected in S–R–H tests were followed by additional post hoc testing using Dunn’s many-to-one method, which is ideal for comparing multiple dose responses to vehicle/control treatments. The false discovery rate was corrected for using the Benjamini, Hochberg, and Yekutieli method. All outliers, main effects, and post hoc analyses are included in Supplementary Files 1–3. In addition, notes regarding injection performance (by the experimenter) were kept during all procedures, and were the primary determinant for other exclusions. To mitigate the effects of repeated testing, such as in the case of animals shown in Fig. 2, data in this figure have been normalized to the responses of vehicle-treated animals that were tested at the same time.

### Reporting summary

Further information on research design is available in the Nature Research Reporting Summary linked to this article.

## Supplementary information

Supplemental Figures

Reporting Summary

## Data Availability

All original data are available upon request of the correspnding author or on the figshare repository with the following link: 10.6084/m9.figshare.12479912^42^.

## Code availability

Standard code was utilized in R (v.1.1.456, RStudio Team); however, custom code was written in MATLAB (r2019a, MathWorks) to organize and discretize time-series data acquired during open-field testing. This code is available upon request to the corresponding author, Nicole M. Ashpole (nmashpol@olemiss.edu).
