# Supplementary material for: Age-dependent hormesis-like effects of the synthetic cannabinoid CP55940 in C57BL/6 mice
Source: NPJ Aging Mech Dis. 2020 Jul 6;6:7. doi: 10.1038/s41514-020-0045-7 (PMC7338393; doi:10.1038/s41514-020-0045-7)

# Supplemental Figure 1 – Experimental Timeline

## Acclimation Phase 1

**Days:** 1, 2, 3

- Weight
- Temperature (IR + Rectal)

## Acclimation Phase 2

**Days:** 4, 5, 6

- Weight
- Temperature (IR + Rectal)
- Vehicle Injection (IP)
- 60m Rest
- **Temperature (IR + Rectal)**

## Testing Battery

**Day:** 7

- Weight
- Temperature (IR + Rectal)
- Vehicle Injection (IP)
- 30m Rest
- **Open Field (30-60m)**
- **60m Temperature (IR + Rectal)**
- **Hot Plate**

# Supplemental Figure 2 - Baseline Measures During Vehicle Acclimation

| Measure | Baseline Weight | | | | Baseline Rectal Temperature | | | | Change in Rectal Temperature | | | | Baseline Skin/Pelage (IR) Temperature | | | | Change in Skin/Pelage (IR) Temperature | | | |
|---|---|---|---|---|---|---|---|---|---|---|---|---|---|---|---|---|---|---|---|---|
| Sex | Male | | Female | | Male | | Female | | Male | | Female | | Male | | Female | | Male | | Female | |
| Age | Young | Old | Young | Old | Young | Old | Young | Old | Young | Old | Young | Old | Young | Old | Young | Old | Young | Old | Young | Old |
| Mean | 27.77 | 32.81 | 22.62 | 27.81 | 37.24 | 36.86 | 37.49 | 36.88 | -0.17 | -0.28 | -0.18 | -0.11 | 30.43 | 29.94 | 30.75 | 29.54 | -0.30 | -0.12 | -0.34 | -0.03 |
| S.E.M. | 0.06 | 0.12 | 0.10 | 0.21 | 0.02 | 0.03 | 0.04 | 0.04 | 0.02 | 0.04 | 0.04 | 0.06 | 0.03 | 0.06 | 0.06 | 0.06 | 0.03 | 0.04 | 0.06 | 0.07 |
| n= | 655 | 510 | 144 | 144 | 655 | 510 | 144 | 144 | 655 | 510 | 144 | 144 | 655 | 510 | 144 | 144 | 654 | 510 | 144 | 144 |

# Supplemental Figure 3 – Effects of CP55940 and AM630 coadministration in Young Males

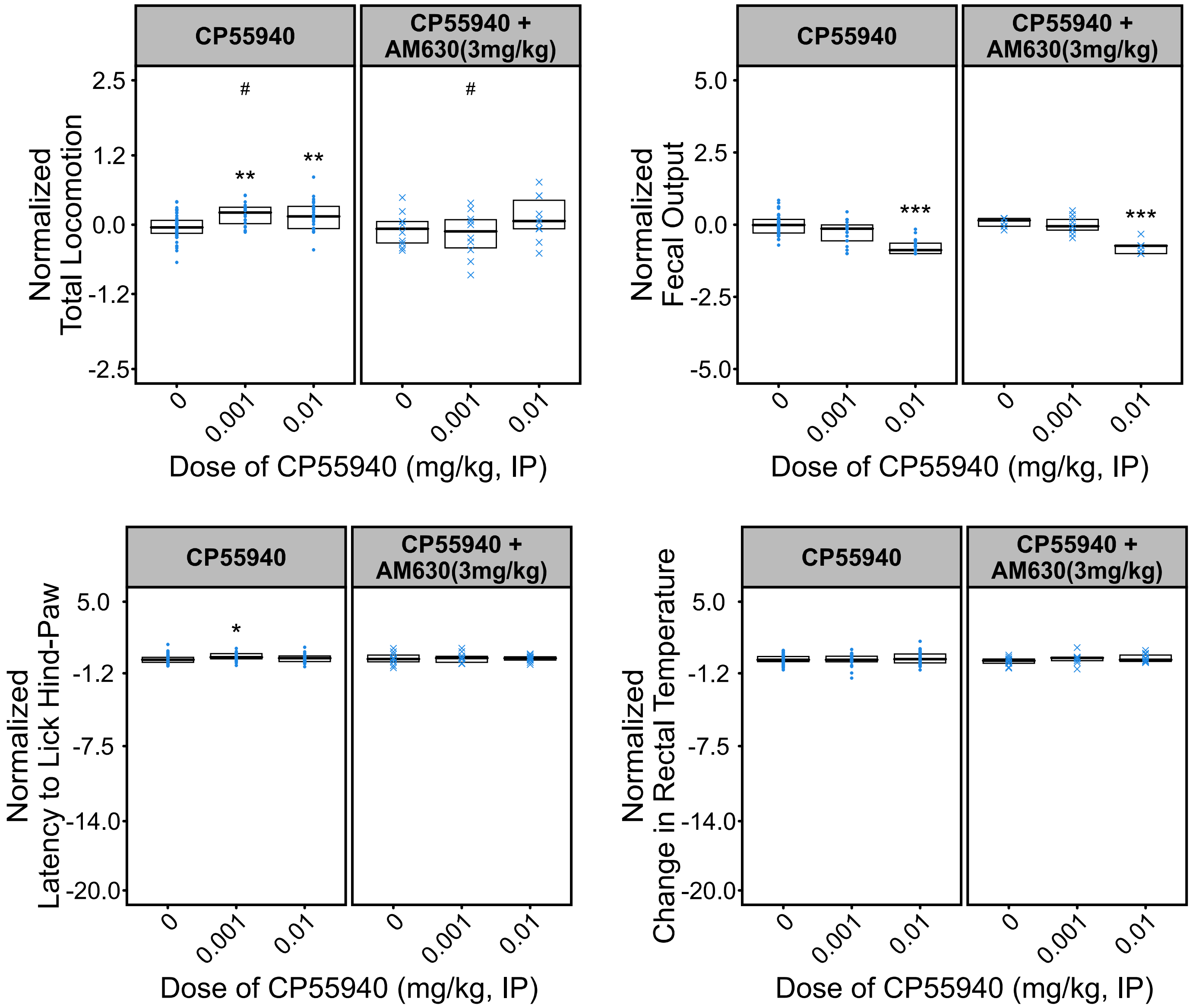

# Supplemental Figure 4 – Animal Cohorts and Experiments

| Total Animals for All Experiments | | |
|---|---|---|
| **Age** | **Sex** | **n=** |
| Young | Male | 125 |
| Young | Female | 28 |
| Old | Male | 60 |
| Old | Female | 21 |

**Data in Figures 2 and 3 are expressed as % of vehicle treated control (tested on same day)**

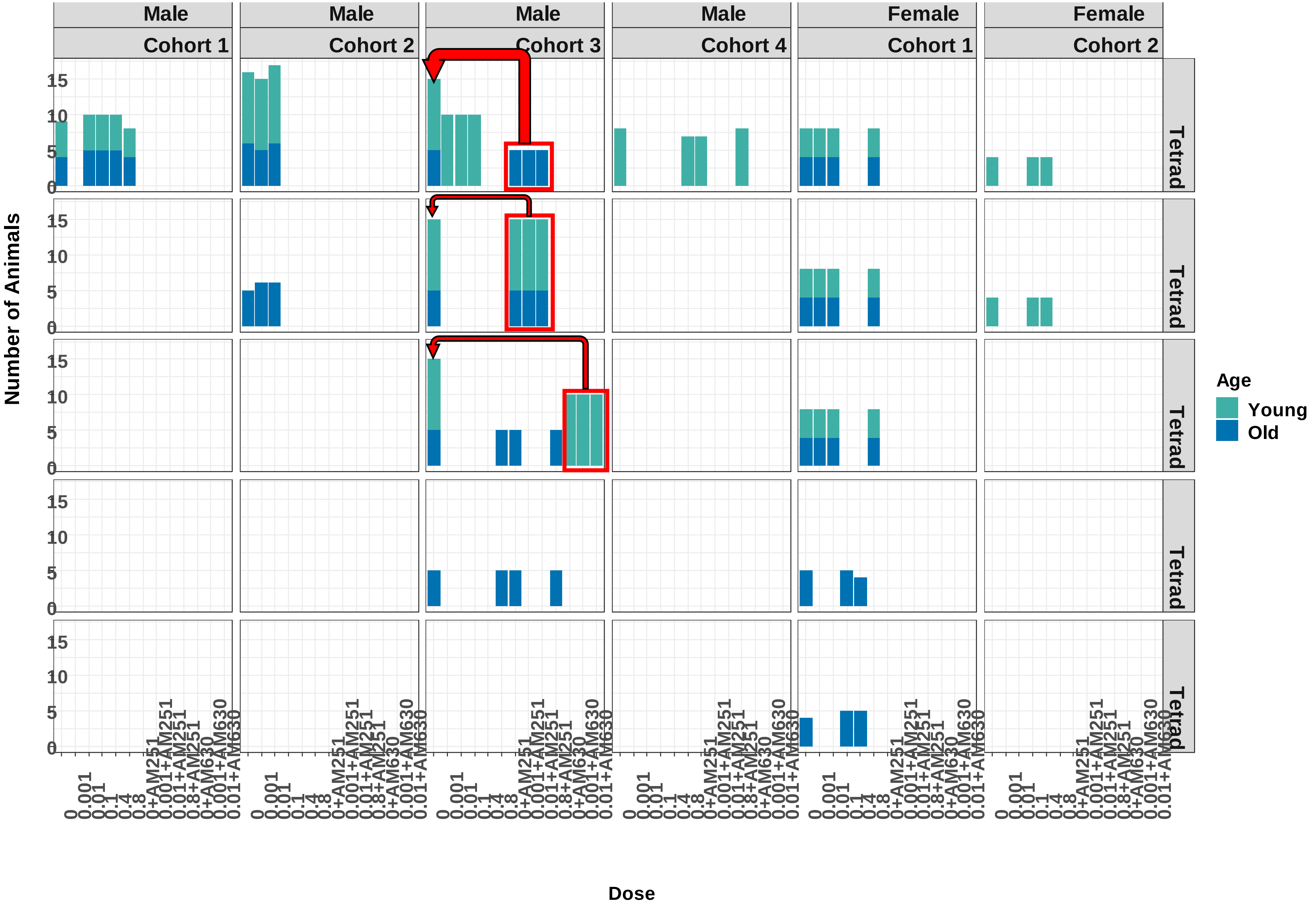

# Supplemental Figure 5 - Sample Sizes

## Sample Sizes in Figure 1

| Dose | Sex | Distance_30m_Total | | Fecal_Output | | Pain_Latency_Hot_Plate | | Pain_Latency_Tail_Water | | Temp_Rectal_Change | | Temp_IR_Change | |
|---|---|---|---|---|---|---|---|---|---|---|---|---|---|
| | | Young | Old | Young | Old | Young | Old | Young | Old | Young | Old | Young | Old |
| 0 | All | 73 | 56 | 68 | 52 | 73 | 56 | 28 | 40 | 73 | 56 | 71 | 56 |
| 0.001 | All | 32 | 23 | 32 | 23 | 32 | 23 | 12 | 12 | 32 | 23 | 32 | 22 |
| 0.01 | All | 38 | 29 | 33 | 24 | 38 | 29 | 12 | 11 | 38 | 28 | 35 | 29 |
| 0.1 | All | 23 | 15 | 15 | 9 | 23 | 15 | 8 | 10 | 23 | 13 | 20 | 15 |
| 0.4 | All | 13 | 13 | 8 | 7 | 13 | 14 | 8 | 9 | 13 | 14 | 11 | 14 |
| 0.8 | All | 22 | 26 | 17 | 21 | 23 | 26 | 19 | 22 | 23 | 25 | 21 | 26 |

## Sample Sizes in Figure 2 / Sample Sizes in Figure 3

| | | Sample Sizes in Figure 2 | | | | | | | | Sample Sizes in Figure 3 | | | | | | | |
|---|---|---|---|---|---|---|---|---|---|---|---|---|---|---|---|---|---|
| Measure | | Distance_30m_Total | | | | Fecal_Output | | | | Pain_Latency_Hot_Plate | | | | Temp_Rectal_Change | | | |
| Dose Group | | CP55940 Alone | CP55940+ AM251 | CP55940 Alone | CP55940+ AM251 | CP55940 Alone | CP55940+ AM251 | CP55940 Alone | CP55940+ AM251 | CP55940 Alone | CP55940+ AM251 | CP55940 Alone | CP55940+ AM251 | CP55940 Alone | CP55940+ AM251 | CP55940 Alone | CP55940+ AM251 |
| Sex | Dose | Young | Young | Old | Old | Young | Young | Old | Old | Young | Young | Old | Old | Young | Young | Old | Old |
| Male | 0 | 51 | 17 | 35 | 20 | 48 | 17 | 31 | 20 | 53 | 14 | 35 | 20 | 53 | 17 | 35 | 18 |
| Male | 0.001 | 20 | 10 | 11 | 10 | 20 | 10 | 11 | 10 | 20 | 10 | 11 | 10 | 20 | 10 | 11 | 10 |
| Male | 0.01 | 26 | 10 | 17 | 10 | 21 | 10 | 12 | 10 | 26 | 10 | 17 | 10 | 26 | 10 | 17 | 9 |
| Male | 0.8 | 10 | 8 | 12 | 10 | 7 | 8 | 10 | 10 | 11 | 8 | 14 | 10 | 11 | 8 | 14 | 10 |

# Supplemental Figure 6
# Effects of CP55940 on Peripheral Locomotion in the Open Field

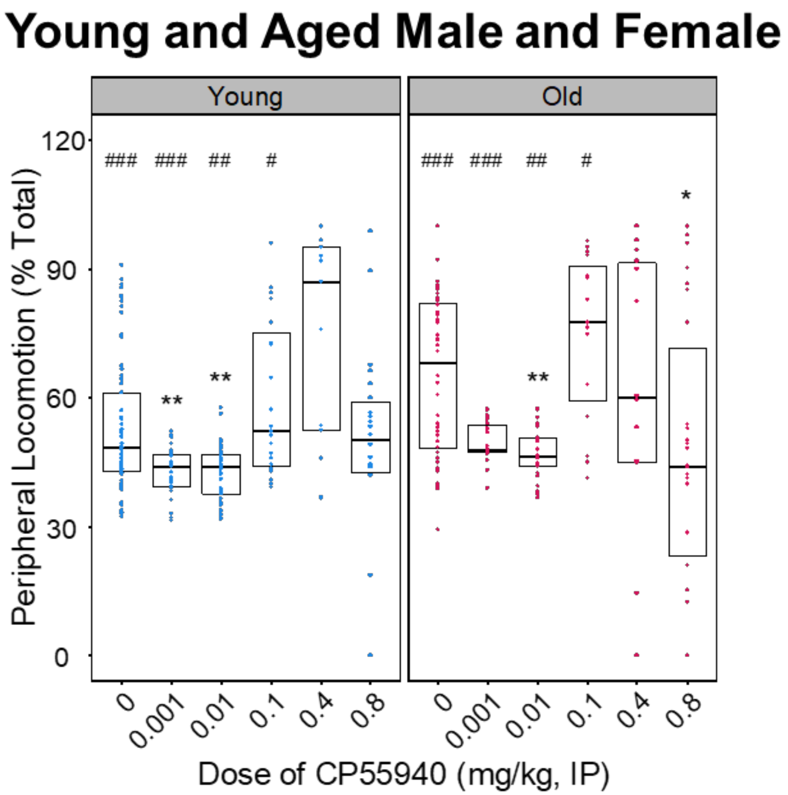


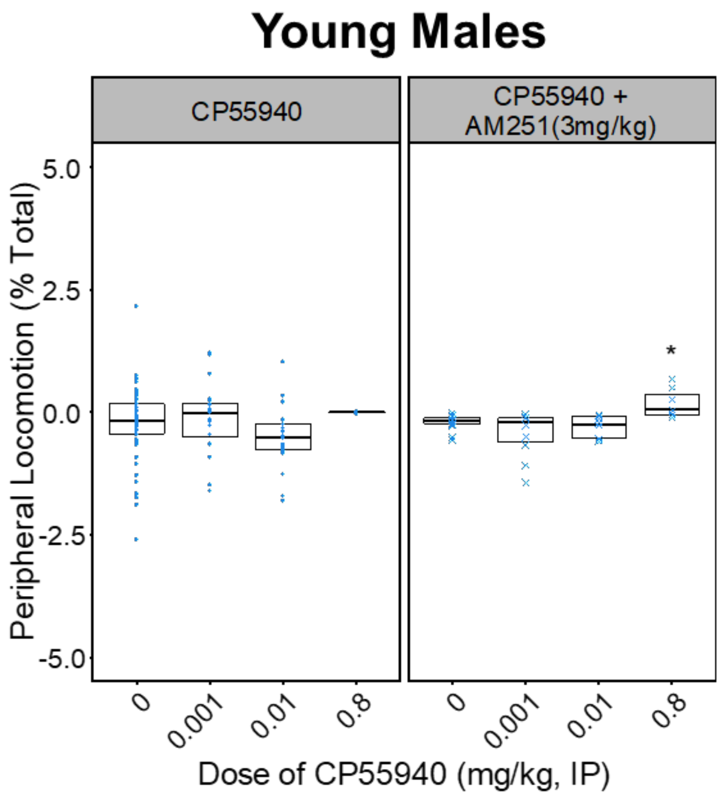

Supplement: Supplementary file 1 — Supplemental Figures [file 41514_2020_45_MOESM1_ESM.pdf]
